# Supplementary material for: Respiratory Function in Friedreich’s Ataxia
Source: Children (Basel). 2022 Aug 29;9(9):1319. doi: 10.3390/children9091319 (PMC9497209; doi:10.3390/children9091319)
Supplement: Supplementary file 1 [file children-09-01319-s001.zip › children-1855478-supplementary.pdf]

Table S1. One-way ANOVA spirometry and degree of correlation with severity of scoliosis.

Variable	Correlation	*p*-Value
FVC sitting	3.84	0.033
FVC sitting % predicted	3.69	0.037
FVC supine	4.47	0.020
FVC supine % predicted	3.83	0.033
fev1 sitting	2.31	0.118
fev1 sitting % predicted	2.31	0.118
fev1 sup	3.23	0.054
fev1 sup % predicted	2.56	0.095
Tif sitting	0.95	0.397
Tif sup	0.39	0.681
Pef	0.56	0.579
Pcf	3.05	0.063
Pef pcf	1.46	0.250
mip	0.32	0.732
mep	0.77	0.471

Figure S1. (**A**) Sitting - supine FVC values expressed as + or − % for each subject; (**B**) % difference expressed as mean, median, SD and range (two outliers, subjects 22 and 27 are marked separately as individual points.

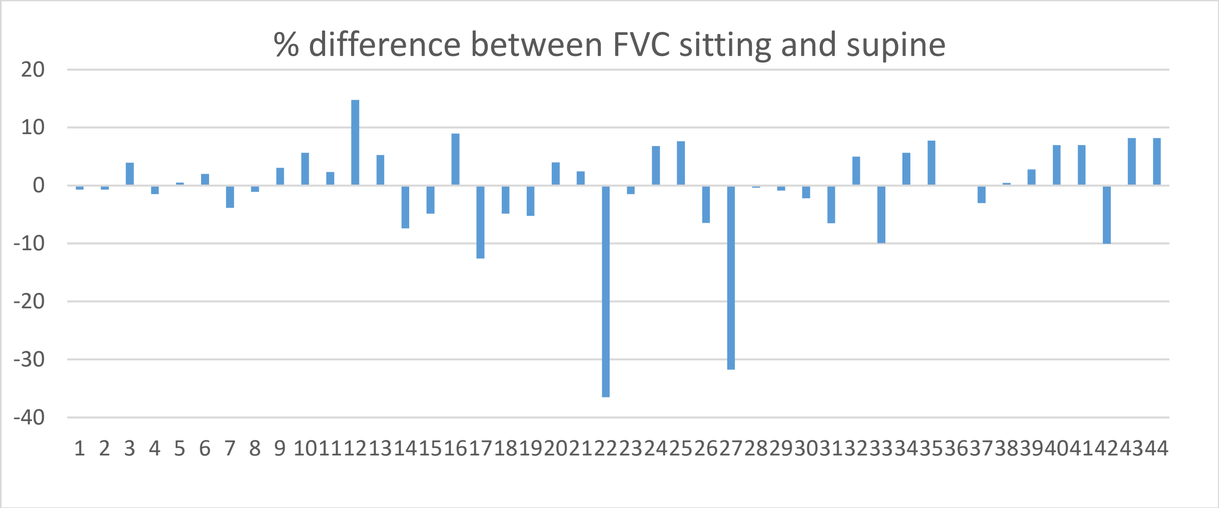

(**a**)

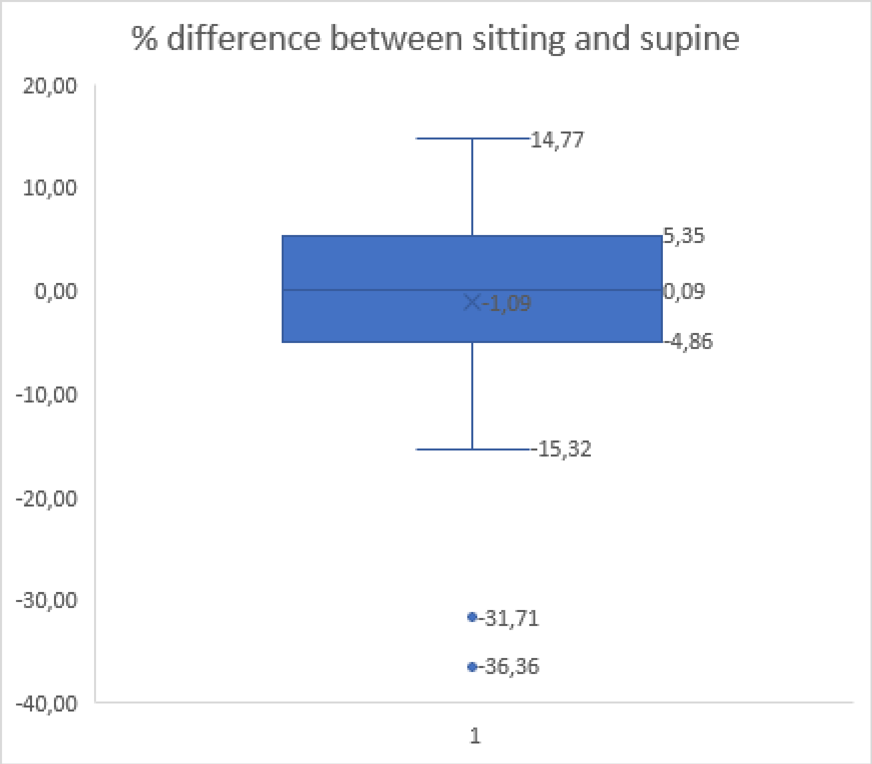

(**b**)
